# Novel Therapeutic Targets and Immune Dysfunction in Malignant Pleural Mesothelioma

**DOI:** 10.3389/fphar.2021.806570

**Published:** 2022-01-07

**Authors:** Moshe Lapidot, Srinivas Vinod Saladi, Ravi Salgia, Martin Sattler

**Affiliations:** ^1^ Department of Thoracic Surgery, Galilee Medical Center, Nahariya, Israel; ^2^ Department of Otolaryngology, Massachusetts Eye and Ear Infirmary, Harvard Medical School, Boston, MA, United States; ^3^ Broad Institute of Harvard and MIT, Cambridge, MA, United States; ^4^ Department of Medical Oncology and Therapeutics Research, City of Hope, Duarte, CA, United States; ^5^ Department of Medicine, Harvard Medical School, Boston, MA, United States; ^6^ Department of Medical Oncology, Dana-Farber Cancer Institute, Boston, MA, United States

**Keywords:** malignant pleural mesothelioma, STAT3, heparanase, SETD2, KDM4A

## Abstract

Advances in the treatment of malignant pleural mesothelioma (MPM) have been disappointing, despite the apparent need for new therapeutic options for this rare and devastating cancer. Drug resistance is common and surgical intervention has brought benefits only to a subset of patients. MPM is a heterogenous disease with a surprisingly low mutation rate and recent sequencing efforts have confirmed alterations in a limited number of tumor suppressors that do not provide apparent insights into the molecular mechanisms that drive this malignancy. There is increasing evidence that epigenetic regulation leads to immune evasion and transformation in MPM. Further, the low efficacy of immune checkpoint inhibitors is consistent with a suppression of genes involved in the anti-tumor immune response. We review three promising emerging therapeutic targets (STAT3, KDM4A, heparanase) and highlight their potential effects on the immune response.

## Introduction

Malignant pleural mesothelioma (MPM) is a rare cancer with a highly malignant and aggressive phenotype that is mainly related to the inhalation of asbestos fibers through occupational exposure. Even though asbestos use has been banned in many countries, there can be still continuing exposure through global and international travel, trade, military-deployments or exposure to asbestos in older buildings. MPM is less frequently associated with non-asbestos fibers, prior radiation exposure and is occasionally idiopathic (Chirieac et al., 2013; Attanoos et al., 2018). It is not know how exactly asbestos causes cancer but chronic inflammation and iron contamination may contribute to oxidative stress and DNA damage, which in turn may also affect the anti-tumor immune response (Matsuzaki et al., 2012). It is thought that MPM has a long latency and thus the incidence increases over time with a median age of 62 years at diagnosis in the United States. Unfortunately, the median overall survival is only 8–12 months. The least aggressive, epithelioid histologic subtypes of MPM is most common compared to the biphasic and sarcomatoid subtypes (Christoph and Eberhardt 2014; Liou and Berry 2018). Metastatic growth is not typical for MPM and the cancer is rather associated with invasive growth of tumor cells and compression of mediastinal structures (Corson and Renshaw 1996; Sugarbaker et al., 1997; Cury et al., 1999).

**FIGURE 1:**
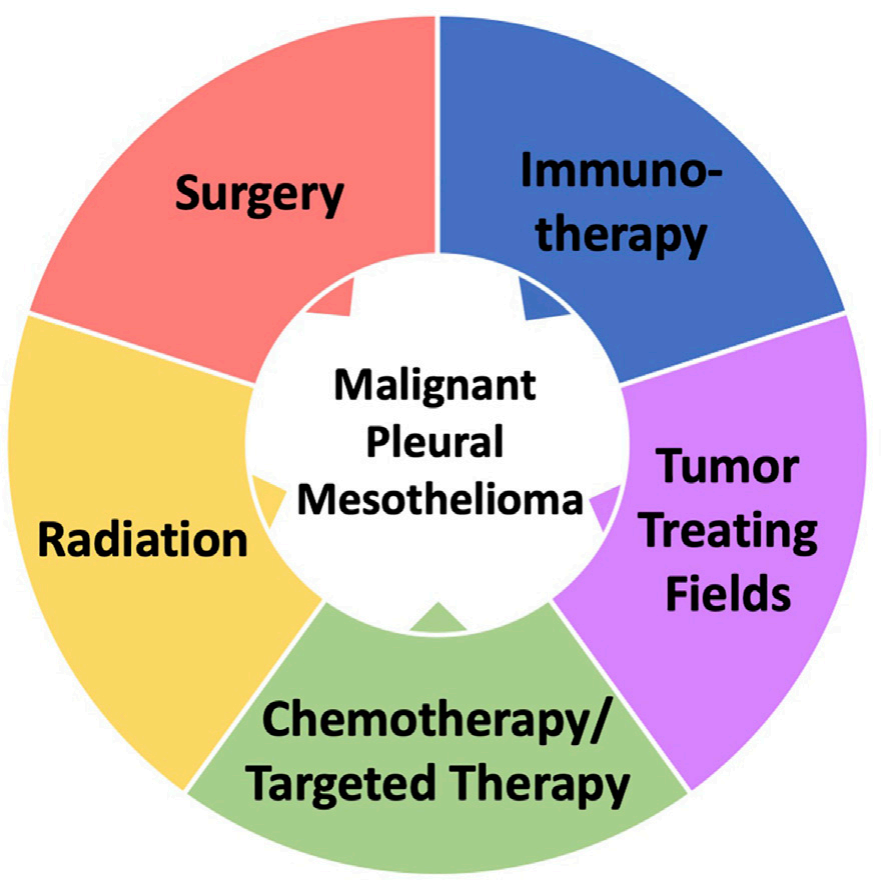
Modalities of MPM Treatment.

In contrast to many other cancers, innovative progress in the treatment of MPM has been slow. The therapeutic options are limited and with an overall poor survival there is an urgent need for novel therapeutic approaches (Cantini et al., 2020). Current therapeutics options for the treatment of MPM can be divided in five modalities, including chemotherapy/targeted therapy, radiation, surgery, immunotherapy and tumor treating fields (Figure 1). With response rates of only about 40%, MPM is often refractory to standard chemotherapy, suggesting that drug resistance is an early concern. Currently, the most effective combination chemotherapy includes the cytotoxic drug cisplatin with the anti-folate pemetrexed (Remon et al., 2015; Wald and Sugarbaker 2018). Targeting VEGF-A with bevacizumab can also be combined with this approach, leading to an additional improvement in median overall survival by 2.7 months (18.8 months total) observed in the MAPS trial (Zalcman et al., 2016). Surgery based multimodality treatment is the main approach for curative intent in MPM patients with resectable disease. Successful cytoreductive surgery (pleurectomy/decortication) in qualified patients can significantly improve survival (Lapidot et al., 2020b). The discovery of biomarkers in epithelioid and biphasic MPM to guide surgical decision and to help predict outcome or disease recurrence after tumor resection holds promise, but has not come to clinical fruition (Gordon et al., 2009). Factors that were found to be associated with longer patient overall survival included epithelioid histology, T stage, quantitative clinical stage/tumor volume staging, adjuvant chemotherapy, intraoperative heated chemotherapy, female sex, and length of stay shorter than 14 days (Lapidot et al., 2020b). Tumor treating fields are a novel devise-based approach that applies alternating electric fields to the tumor and directly affects molecules with dipole characteristics, thus disrupting normal cell division. Tumor treating fields can also be combined with standard chemotherapy although definitive evidence of efficacy is lacking (Ceresoli et al., 2019). Nevertheless, the impact of tumor treating field therapy on clinical outcomes may need further evaluation due to the design of the original study and its comparison of clinical data with historical responses to chemotherapy. More recently, immunotherapy using immune checkpoint inhibitors has also shown some success in MPM. Combination of nivolumab (anti-PD-1) plus ipilimumab (anti CTLA-4) is efficacious, in particular in MPM patients with non-epithelioid phenotype, extending the median overall survival by 9.3 months (18.1 months total) in this group or by 4 months for all patients (Baas et al., 2021). Current clinical trials build upon improving this approach and there is a particular urgency to identify patients that would benefit from this treatment. Cancer immunotherapy holds great promise and has become an exciting tool in the treatment of MPM, but careful analyses of recent approaches suggest that progress is rather incremental and some clinical trial data may require a closer and more unbiased look (Correale et al., 2021). Overall, the dismal prognosis in MPM is caused by a paucity of potential therapeutic targets and the lack of efficacious long-term treatments. The past characterization of the genomic landscape in MPM may not be sufficient to draw conclusions for future drug development as there is a significant patient-specific variability in mutations. Sucessful approaches may need a more robust biomarker approach that brings MPM closer to personalized medicine.

## Malignant pleural Mesothelioma is Defined by Tumor Suppressors

**FIGURE 2:**
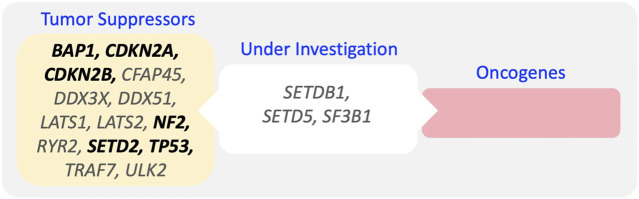
Frequent Mutations in MPM. Genes with validate tumor suppressor function, and not-classified genes are indicated (genes found with a mutation frequency >5% are indicated in bold).

The genetic changes in MPM have been well defined and abundantly described in the literature. Chromosomal abnormalities and whole exome sequencing have allowed mapping of these changes and revealed a surprising paucity of actionable targets, such as mutationally activated oncogenes. Consequently, the vast majority of recurrent mutations predominantly result in loss-of-function of tumor suppressors (Figure 2), including BAP1, TP53, NF2, SETD2, LATS2, DDX3X, and ULK2, as well as copy number losses in BAP1, TP53, CDKN2A/B, NF2, LATS2, LATS1 and gains in RPTOR and BRD4 as well as other genomic alterations (Bueno et al., 2016; Hmeljak et al., 2018). Somehow, in particular small chromosomal deletion are often not covered in these analyses. Whereas all these change are not necessarily concurrent, they give interesting insights into the mechanisms of transformation that drive MPM. However, none of these mutations are disease-specific, such as SETD2 mutations, which have also been found in other malignancies (Fahey and Davis 2017).

Changes in the histone methyl transferase SETD2 are of particular interest in the context of MPM, as they hint directly at the fact that the disease is driven by epigentic changes. SETD2 is required for trimethylation of histone H3 lysine 36, whereas the mono- and dimethylation of lysine 36 is regulated by other methyltransferases (Edmunds et al., 2008; Yuan et al., 2009). It would thus be interesting to correlate changes in histone methylation marks to the mutational status and gene expression patterns. A closer look at individual alterations in mesothelioma patients indicates frequent biallelic deletions of gene clusters involving BAP1, SETD2, SMARCC1 and PBRM1 on chromosome 3p21 (Yoshikawa et al., 2016). These changes bear striking similarities to changes in renal cell carcinoma where these molecules are centrally involved in dysregulating functions related to the SWI/SNF (SWItch sucrose non-fermentable) chromatin remodelling complexes leading to a broad change in epigenetic mechanism mediated gene expression (de Cubas and Rathmell 2018). BAP1, is a prominent mutational target in MPM that reduces histone H2A lysine 119 mono-ubiquitination, thereby reducing the PRC1 (polycomb repressor complex 1) function and moving the related PRC2 away from its targets (Conway et al., 2021). Genetic models suggest an epigenetic antagonism between Polycomb and SWI/SNF (Wilson et al., 2010) and BAP1 would therefore predicted to effectively change this balance of epigenetic factors.

As mentioned above, the mutational landscape is not a reflection of individual changes in tumors and does not provide any information about tumor evolution, which would be helpful to understand critical changes. A recent study by Zheng and colleagues analyzing 90 tumor samples from 22 MPM not only highlights the exomic intratumour heterogeneity within MPM, but also identifies early genetic changes associated with transformation in MPM (Zhang et al., 2021). Initial events included allelic heterogeneity associated with BAP1 (deubiquitinates histone H2A) or its locus 3p21 and frequent mutations of FBXW7 (4q31.3) (phosphorylation-dependent ubiquitination) or loss of chromosome 4, whereas NF2 mutations or loss of 22q (hippo pathway) were late events. The recurrence of these mutations demonstrate constraints in the tumor evolution that indicate vulnerabilities and may provide future therapeutic targets. The changes have implications for immunotherapy approaches as genomic instability in MPM was associated with increased T-cell infiltration and immune escape though immunoediting via HLA loss of heterozygocity. This would allow MPM cancer cells to escape T-cell mediated response in the presence of higher neoantigen burden, but it also links mutational changes to alterations in the tumor microenvironment (Zhang et al., 2021). Future clinical trials using immune checkpoint inhibitors may therfore not only require a careful analysis of immune markers as previously suggested (Awad et al., 2016), but may also need a more careful analysis of the mutational status of patients’ tumors.

## Emerging Targeted Therapies

**FIGURE 3:**
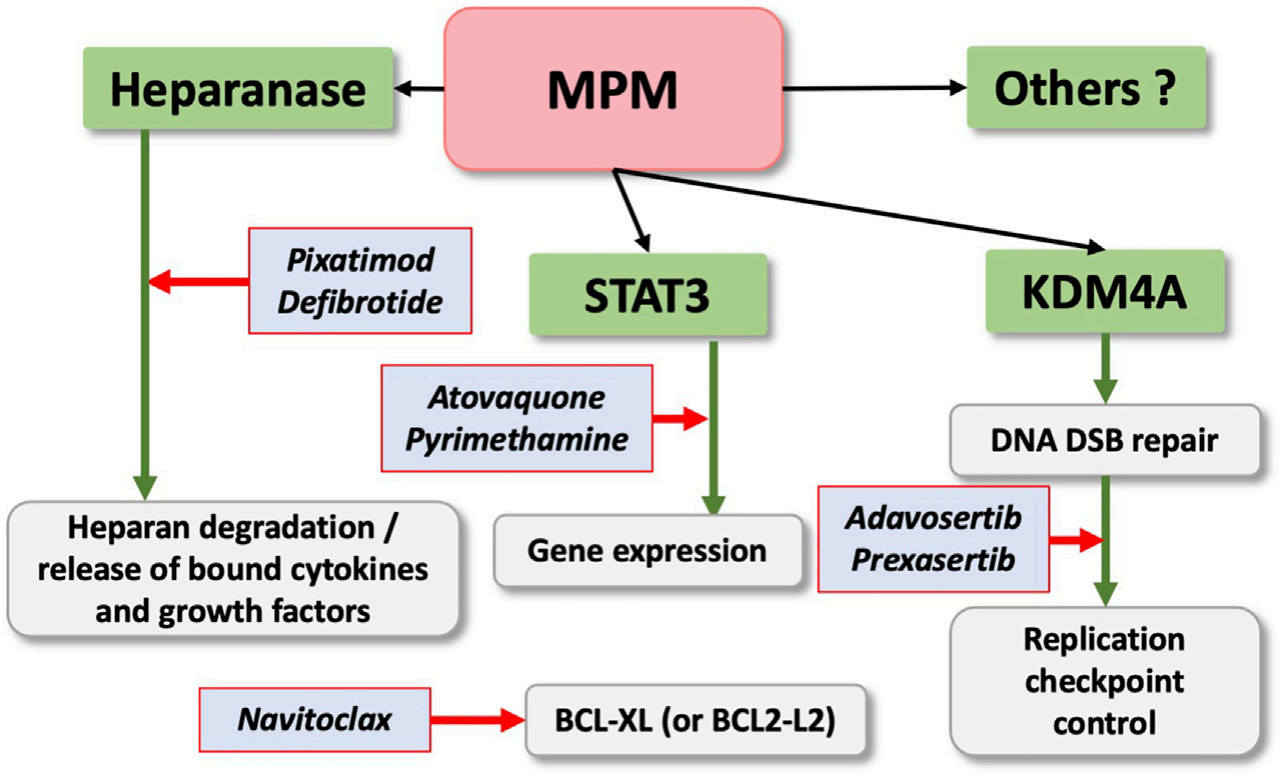
Emerging Therapeutics Targets in MPM. STAT3, KDM4A, heparanase are promising new thereapeutic targets in MPM. Clinical stage inhibitors are indicated and treatment may benefit from combination with navitoclax.

The development of novel and effective targeted therapies in MPM has been disappointing over the past decade. Recent identification of a variety of potential new targets for drug development is encouraging (Cantini et al., 2020). One current goal is to combine immunotherapy with targeted therapeutics to overcome cancer-induced immune evasion. We describe three novel promising therapeutic targets, including STAT3, KDM4A and heparanase, that show significant efficacy in pre-clinical models of MPM with immune implications (Figure 3). Frequent mutations in mesothelioma have not yet been linked to a common signaling pathway. However, it has become clear that histone modifications and chromatin remodeling involving nuclear histones play a central role in the activation of signaling processes and malignant transformation. This is supported by frequent mutations in the BAP1 tumor suppressor, which mono-ubiquitinates histone H2AK119 and therefore regulates Polycomb Repressive Complex 1 (PRC1)-mediated repression of genes [see for review (Szczepanski and Wang 2021)]. The function of STAT3 can be in part controlled by the BRG1 (SMARCA4) core subunit of the SWI/SNF chromatin remodeling complex through direct binding and co-transcriptional regulation (Zhu et al., 2016); KDM4A demethylates the histone H3 methylation mark on lysine 9 and 36 (Klose et al., 2006); and heparanase can regulate chromatin remodeling by increasing the levels of acetylated histone H3, expected to result in altered gene expression (Amin et al., 2020). Heparanase expression itself can be controlled by the helicase-like transcription factor (HTLF), a member of the SWI/SNF family of proteins (Dhont et al., 2016). The involvement of SWI/SNF and other chromatin remodeling complexes in MPM has not been carefully evaluated and requires an in-depth analysis to gain a better understanding of their exact contributions to the disease process. Clinical stage inhibitors (drugs in clinical trials) or approved drugs are available and future clinical trials are expected to test their efficacy in MPM. There are additional promising approaches at various pre-clinical or clinical stages that require careful consideration as well.

### The STAT3 Transcription Factor Regulates Growth and Immune Response Genes

#### STAT3 is Frequently Activated in Malignant Pleural Mesothelioma

STAT3 (Signal Transducer and Activator of Transcription 3) is a transcription factor that is normally transiently activated after stimulation of certain cytokine or growth factor receptors, where it controls genes involved in regulating survival, proliferation and self-renewal (Galoczova et al., 2018; Roca Suarez et al., 2018). In many solid tumors and hematologic malignancies, dysregulated STAT3 activation is a central event and drives malignant transformation, often through activation of tyrosine kinases (Pencik et al., 2016). In contrast to normal signaling, in many malignancies STAT3 has oncogenic features and regulates genes that are involved in critical cellular processes, including survival, self-renewal, proliferation, angiogenesis and invasion (Pencik et al., 2016; Guanizo et al., 2018). The rate of STAT3 activation in MPM is high and tyrosine phosphorylated STAT3 was found in 61.4% (27/44) of archived cases (Achcar Rde et al., 2007). This number could even be higher as phosphorylation sites in archived samples may not be stable. The mutational landscape in MPM does not provide direct evidence for the activation of STAT3 (Bueno et al., 2016; Hmeljak et al., 2018) and epigenetic regulation may play an important role in its regulation. In MPM, tyrosine kinases are rarely mutationally activated, but we have previously demonstrated that STAT3 can be infrequently activated by EPHA2 mutations with oncogenic characteristics (Tan et al., 2019). Low expression levels of PIAS3 (Protein inhibitor of activated STAT3) may also be associated with increased STAT3 activation and poor survival in MPM (Dabir et al., 2014), but low expression of STAT3 does not exclude dependency on this pathway (Lapidot et al., 2020a).

#### STAT3 is Required for Optimal Growth

We and others have previously shown that active STAT3 is required for growth of MPM cells (Abdul Rahim et al., 2015; Lapidot et al., 2020a). STAT3 was not only required for optimal growth of these cells but was also sufficient to enhance the growth of the LP9 mesothelial cell line (Lapidot et al., 2020a). We demonstrated the efficacy of the STAT3 pathway inhibitors atovaquone (Xiang et al., 2016) and pyrimethamine (Takakura et al., 2011; Khan et al., 2018) using *in vitro and in vivo* models of MPM (Lapidot et al., 2020a). These drugs have been approved for other indications and can easily be repurposed. This approach has the potential to have a reasonable therapeutic index, since normal tissue is less dependent on STAT3 activation. In addition, for surgical candidates, it might be possible to administer drugs in the pleural space, which would allow to achieve even greater therapeutic effects. Despite its apparent role in other cancers, the function of STAT3 in MPM is poorly defined. In MPM regulators of apoptosis and cell cycle, including TP53 (encoding for p53) and CDKN2A (encoding for p16INK4A and p14ARF) are frequently mutated and render cells resistant to apoptotic stimuli (Hernandez-Monge et al., 2016; Hmeljak et al., 2018). This may be in part through loss of the regulatory function of p53 on STAT3, resulting in increased STAT3 activity (Schulz-Heddergott et al., 2018). Also, a functional and physical interaction between STAT3 and NFkB may be required for chemoresistance in MPM (Canino et al., 2015).

#### STAT3 Contributes to Immune Dysfunction

Also, immunoediting and escape from attack by the immune system is a major hallmarks of malignant transformation (Hanahan and Weinberg 2011) and despite some success with immune checkpoint inhibitors, MPM is generally considered to be an immunologically “cold” tumor. Targeting STAT3 gene expression may be beneficial by enhancing immune effector function and decreasing immune evasive mechanisms. Targeting the STAT3 pathway was found to upregulate of CXCL8 (interleukin-8) and ICOSLG (Inducible T-Cell Costimulator Ligand or B7H2) (Lapidot et al., 2020a). Interleukin-8 has the potential to retain antigen-presenting dendritic cells in the tumor (Feijoo et al., 2005) and it may contribute to tumor growth in MPM in at least one murine model (Galffy et al., 1999; Alfaro et al., 2017). ICOS-L (ICOS-ligand), a co-stimulatory B7 family member (B7H2), interacts with the ICOS receptor on T cells and is likely to support anti-CTLA-4 immune checkpoint therapy (Fu et al., 2011). There are additional molecules regulated by the STAT3 pathway inhibitors pyrimethamine and atovaquone strongly supporting a growth promoting and immune suppressive role of STAT3 in MPM (Lapidot et al., 2020a) and therefore supporting a role of STAT3 as a promising therapeutic target.

### KDM4A has Oncogenic Features in Malignant Pleural Mesothelioma

#### Regulation of Histone Methylation by SETD2 and KDM4A

Loss-of-function mutations in the *SETD2* tumor suppressors that encodes for an H3 histone H3K36 lysine N-methyltransferase, are frequently found in MPM. Its activity is in part balanced by the lysine-specific histone demethylase 4 (KDM4) enzymes that not only demethylates the SETD2 product H3K36me3 but also H3K9me3. The latter function of KDM4 family members is thought to primarily regulate the majority of its biological activity (Young and Hendzel 2013; Guerra-Calderas et al., 2015). KDM4A reduces levels of dimethylated lysine K36 as well as K9 of histone 3, with a higher affinity for the latter residue (Young and Hendzel 2013), which is thought to regulate self-renewal, DNA repair, splicing, apoptosis, and other biological activities (Monaghan et al., 2019). Our recent data demonstrated that in particular KDM4A is expressed at high levels in MPM patients and that the expression itself is associated with cell growth *in vitro* and *in vivo*, using small molecule drugs and RNA interference (Lapidot et al., 2021). Similar activities have been found in other cancers and KDM4A inhibitors were shown to regulate growth through induction of apoptosis and inhibition of cell cycle progression (Wang et al., 2013; Franci et al., 2017). However, highly specific KDM4A small molecule inhibitors that are suitable for future clinical trials are not yet available and are a focus of current research.

#### KDM4A Dependency and Pathway Vulnerabilities

It is likely that KDM4A pathway dependencies in MPM cells trigger aberrant activities and extend beyond normal function (Young and Hendzel 2013; Guerra-Calderas et al., 2015). However, there are differences between MPM models and the reliance on KDM4A activity within these models is not a universal event. In the absence of clinically relevant KDM4A inhibitors, targeting pathways dependent on or altered by KDM4A may be a viable alternative. These pathways may not necessarily be directly associated with cell growth. For example, in cancer cells with loss of SETD2, a decrease in levels of the RRM2 ribonucleotide reductase subunit can create a vulnerability, which can be exploited by the WEE1 inhibitor adavosertib that further reduce RRM2 levels, leading to S phase arrest as a result of a depleted dNTP pool (Pfister et al., 2015). Similarly, KDM4A regulates RRM2 levels and MPM cells display adavosertib dependent growth in the absence of SETD2 loss (Lapidot et al., 2021). Interestingly, inhibition of WEE1 by adavosertib cooperated with inhibition of CHK1 by prexasertib (Lapidot et al., 2021), another DNA damage and replication checkpoint regulator within the DNA double-strand break repair pathway. This is likely through forcing cells into abnormal mitosis, resulting in subsequent cell death (Aarts et al., 2012). The degree of dysregulation of the DNA double-strand break repair pathway itself in MPM is not well understood though. The data not only support an essential role for KDM4A in cell growth and DNA damage repair but also inversely mirror previously described opposite functions of SETD2 (Young and Hendzel 2013; Fahey and Davis 2017). Finally, the BH3 mimetic navitoclax enhanced KDM4A inhibitor-induced apoptosis, suggesting an independent role for this drug in combination with pro-apoptotic reagents (Lapidot et al., 2021). KDM4A may represent a growing class of mechanisms with oncogenic features that are normally associated with mutated and activated oncogenes. Interestingly, our data also implicate KDM4A in the suppression of HLA expression, which is reminiscent of the loss of heterozygocity in MPM specimen with high tumor burden, further supporting immune evasion mechanisms of the cancer cells (Lapidot et al., 2021; Zhang et al., 2021).

### Heparanase

#### Heparanase Activity Promotes Tumor Growth in Malignant Pleural Mesothelioma

The extracellular matrix (ECM) is essential for tissue integrity and homeostasis. Heparan sulphate is an important component of the ECM by contributing to maintenance of its structural integrity and regulatory functions in the form of heparan sulphate proteoglycans (HSPGs) (Jayatilleke and Hulett 2020). Heparanase, the sole heparan sulfate degrading endoglycosidase, regulates multiple biological activities that enhance tumor growth, angiogenesis and metastasis. Heparanase accomplishes this by degrading heparan sulphate and thereby facilitating cell invasion and regulating the bioavailability of heparin binding proteins. Heparanase expression is enhanced in almost all cancers examined including various carcinomas, sarcomas and hematological malignancies (Vlodavsky et al., 2016). Studies provide compelling evidence that ties heparanase levels with all steps of tumor formation including tumor initiation, growth, metastasis, and chemoresistance (Boyango et al., 2014; Shteingauz et al., 2015). In pre-clinical *in vivo* mouse models of MPM, the dependency on heparanase for tumor growth was demonstrated in the context of heparanase gene disruption and in response to heparanase-inhibiting compounds (Barash et al., 2018; Lapidot et al., 2018). Clinically, patients with high heparanase immunostaining survived less than patients with low levels of heparanase. The clinical results are supported by the ability of heparanase inhibitors to prominently restrain the growth of mesothelioma tumor xenografts implanted orthotopically. Notably, the heparanase inhibitors pixatimod (PG545) and defibrotide appeared more effective than cisplatin, a common chemotherapeutics in mesothelioma, in restraining tumor growth, closely associating with a profoundly prolonged survival of mesothelioma-bearing mice.

#### Heparanase Affects Localization of Tumor Macrophages

Macrophages are a cellular constituent of the tumor microenvironment and form a significant portion of tumour-associated immune cells, with heparanase playing a key role in their activation and function (Aras and Zaidi 2017). Macrophages are capable of promoting tumors through the induction of immunosuppression (Noy and Pollard 2014). Interestingly, in mesothelioma models, although neither the total number of macrophages attracted to tumors nor their classification into M1 or M2 type was affected by pixatimod (PG545), their localization was altered. Accordingly, while macrophages were noted to populate the entire tumor mass in control mice, they appeared to accumulate at the tumor periphery in heparanase-KO mice or upon treatment with pixatimod (PG545) or defibrotide, suggesting that heparanase is required for macrophages to penetrate tumors. Given the pro-angiogenic properties of macrophages, their elimination from the tumor mass may add another explanation for the observed impaired angiogenesis in heparanase-KO mice or following pixatimod (PG545) treatment. It appears that heparanase is a master regulator of the aggressive phenotype of malignant mesothelioma, an important contributor to the poor outcome of mesothelioma patients and a prime target for therapy, encouraging clinical examination of heparanase inhibitors as a new therapeutic modality in mesothelioma.

## Immune Dysfunction and Immunotherapy

### Immune Checkpoint Inhibitors in Malignant Pleural Mesothelioma

**FIGURE 4:**
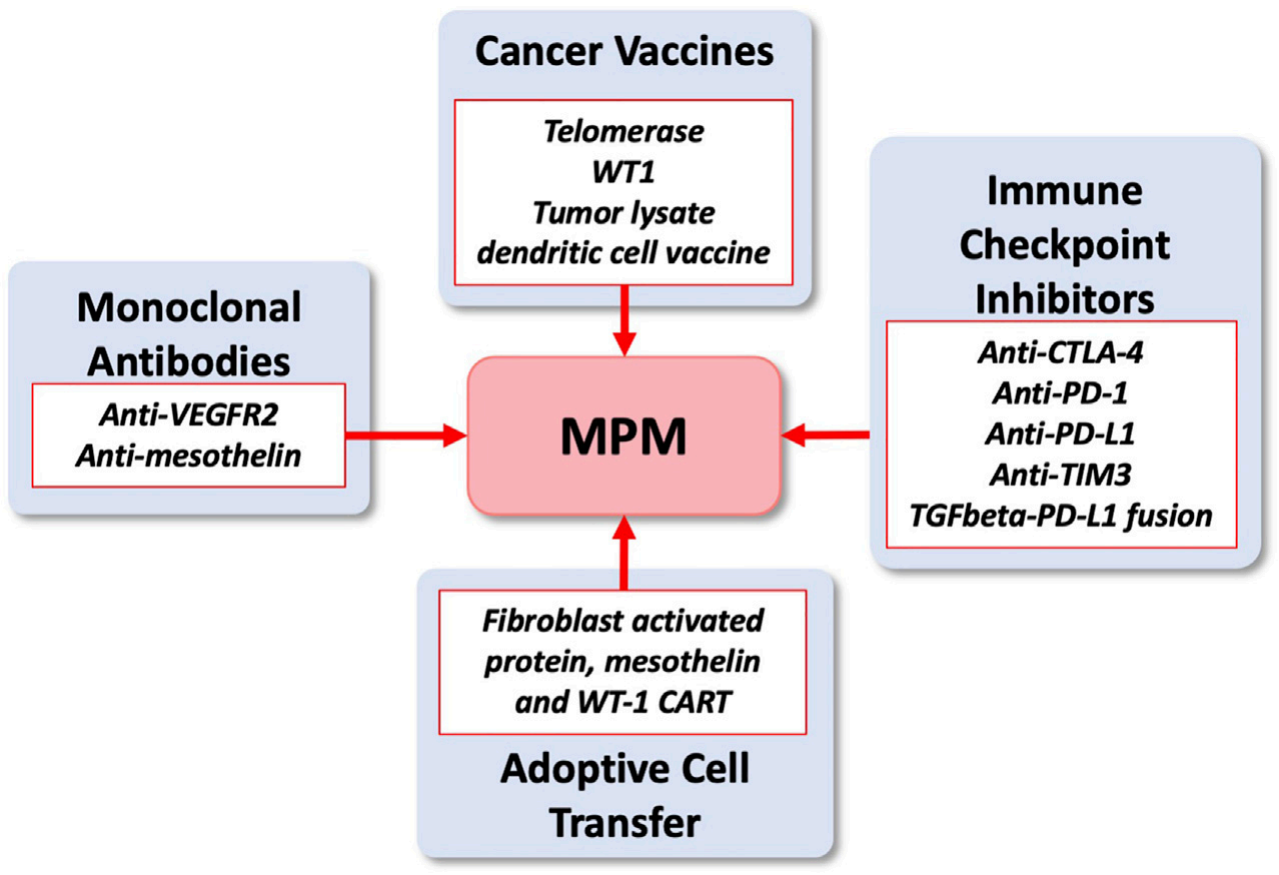
Immunotherapy treatments under investigation in MPM. Ongoing and planned clinical trials in MPM (see clinicaltrials.gov).

The advent of immunotherapy approaches for MPM has started an exciting new phase in the treatement of this disease. Clinical trials focus on the development of immune checkpoint inhibitors, cancer vaccines, monoclonal antibodies and adoptive cell transfer [see for review (Nicolini et al., 2019; Cantini et al., 2020) and clincaltrials.gov] (Figure 4). The combination of nivolumab and ipilimumab is the first FDA approved immunotherapy and first new drug therapy for unresectable MPM patients. The Checkmate 743 trial in 2021 demonstrated significant improvement in overall survival versus standard of care chemotherapy especially for non-epithelioid histology (Baas et al., 2021). The randomised phase 3 CONFIRM trial for patients with relapsed peritoneal and pleural mesothelioma following platinum-based doublet chemotherapy also showed longer progression-free and overall survival with nivolumab alone compared with placebo (Fennell et al., 2021). This is in contrast to the earlier placebo-controlled randomized phase 2 DETERMINE trial of tremelimumab (anti-CTLA-4) in patients with relapsed mesothelioma, where no effect on overall survival was observed (Maio et al., 2017). The challenge is to identify appropriate approaches to turn the immunological ‘cold’ MPM tumor into a tumor with sufficient infiltration of immune cells that can effectively attack the target cells. The anti-PD-L1 (programmed cell death ligand 1) immune checkpoint inhibitor pembolizumab did not improve overall survival in MPM patient compared to single agent chemotherapy (gemcitabine or vinorelbine) in relapsed patients, even though some activity was observed (Popat et al., 2020). It is possible that combinations may be required to efficiently activate T-cells in MPM as observed for the anti-PD-1 plus anti-CTLA-4 therapy. Clinical trials targeting TIM3 are planned (NCT03652077), but there are currently no active trials involving targeting of the immune checkpoint inhibitors VISTA, LAH-3, OX40/OX40L or B7H3. Targeting of VISTA (V-domain Ig Suppressor of T-cell Activation) would be of interest for patients with epitheliod phenotype, which did not fare as well as the non-epithelioid group in the checkmate 743 trial, since the expression is high in this population (Chung et al., 2020).

### Potential for Immuno-Oncology Approaches

Our data show that the expression of OX40L (TNFSF4) is enhanced in response to STAT3 pathway inhibitors (Lapidot et al., 2020a), which may be an opportune target in addition to CTLA4, as mentioned above. Another way to improve the T-cell response are cell based therapies using dendritic cell cancer vaccines or CAR-T cells (Belderbos et al., 2020). The goal of the vaccines is to increase presentation of antigens that are abundantly expressed on MPM cells and therefore increase the pool of specific T-cells, such as those targeting telomerase (UV1), WT1 or whole tumor antigens and therefore enhance the T-cell response. CAR-T cells are modified T-cells that specifically altered to respond to the same or related tumor antigens (WT-1, mesothelin or FAB) on their target cells. Both approaches have limitations and the technologies are still at an evolving stage with variable success. The monoclonal antibody therapies are different as the antibody drug conjugateds (ADCs) deliver toxins directly to the cancer cells, primarily independent of cellular T-cell function. One of the challenges with immunotherapy is the above mentioned poor infiltration of MPM tumors with immune cells (see for review (Chu et al., 2019)). There is also considerable heterogeneity in the tumor immune microenvironment, reflecting the challenges of identifying the right approach to overcome T-cell anergy (Lee et al., 2018). Emerging evidence suggests that tumor immune evasion is a dominant mechanism that specifically selects for certain mutations, such as in tumor suppressors (Martin et al., 2021), hinting at the possibility that many effective targeted therapies may partially revert immune phenotypes. These changes may be highly specific and could have implications for targeted approaches. For example, mutations in the tumor suppressor NF2 compared to those within its putative Hippo-YAP effector pathway define distinct MPM subsets that lead to distinct changes in signaling and immune signatures (Yang et al., 2021). YAP pathway mutations were described in this study to lead to pathway dysregulation and an exhausted CD8 T cell-mediated immunity with upregulated PD-L1, but the same was not necessarily true for loss of NF2. Current single-cell transcriptome approaches will further define these results but may likely provide limited information about which patient populations will benefit from immune checkpoint inhibition without proper correlation to clinical trials. An alternative method to overcome immune evasion would be to directly target dysfunctional immune cells in the tumor microenvironment. For example, functionally indolent tumor infiltrating lymphocytes (TILs) isoated from MPM tissue that were treated with the BTK tyrosine kinase inhibitor ibrutinib and the mTOR kinase inhibitor rapamycin showed downregulating of exhaustion markers and reprogramming of effector memory T cells (TEM) towards terminally differentiated effector memory T cells (TEMRA) (Yang et al., 2022). The interactions of cells within the tumor microenvironement and tumor cells are complex and past experience with other solid tumors have not provided reliable guidance for the use of these therapeutics. However, they may hint at additional approaches that have not yet been considered.

## Conclusion

The lack of highly effective treatment options in MPM is a multilayered problem that may need to be addressed as a whole. MPM is unfortunately often diagnosed at a later stage and currently there are no effective biomarkers that allow easy screening of at-risk patients with a history of asbestos exposure or other inciting events, thus preventing early broad successful intervention. There are no good biomarkers that allow to identify patients likely to respond to current therapies. Tailoring the right treatment approach for each individual patient should be the goal for MPM treatment. Considering the intratumor heterogeneity in MPM, this may not be an easy task and a considerable amount of research will be needed to reach this goal. Single-cell sequencing will be an important tool to better understand the mechanism of immune evasion in MPM and define the immune populations that support tumor growth. Attempts to suppress the immune evasive characteristics of MPM through targeted approaches in combination settings would add additional complexity but would also be predicted to ultimately lead to improved survival. Changes in tumor growth and immune phenotypes in response to molecules such as STAT3, KDM4A or heparanase already demonstrate unique responses that depend on their respective activities. Potential combinations of immunotherapeutics with inhibitors of these pathways would have to be carefully considered, depending on the resulting immune phenotype. Past experience with targeted approaches and immunotherapeutics has shown that there will unlikely be a one-fit-all approach.

## References

[B1] Aarts M. Sharpe R. Garcia-Murillas I. Gevensleben H. Hurd M. S. Shumway S. D. (2012). Forced Mitotic Entry of S-Phase Cells as a Therapeutic Strategy Induced by Inhibition of WEE1. Cancer Discov. 2, 524–539. 10.1158/2159-8290.CD-11-0320 22628408

[B2] Abdul Rahim S. N. Ho G. Y. Coward J. I. (2015). The Role of Interleukin-6 in Malignant Mesothelioma. Transl. Lung Cancer Res. 4, 55–66. 10.3978/j.issn.2218-6751.2014.07.01 25806346 PMC4367717

[B3] Achcar Rde. O. Cagle P. T. Jagirdar J. (2007). Expression of Activated and Latent Signal Transducer and Activator of Transcription 3 in 303 Non-small Cell Lung Carcinomas and 44 Malignant Mesotheliomas: Possible Role for Chemotherapeutic Intervention. Arch. Pathol. Lab. Med. 131, 1350–1360. 10.1043/1543-2165(2007)131[1350:EOAALS]2.0.CO;2 17824789

[B4] Alfaro C. Sanmamed M. F. Rodríguez-Ruiz M. E. Teijeira Á. Oñate C. González Á. (2017). Interleukin-8 in Cancer Pathogenesis, Treatment and Follow-Up. Cancer Treat. Rev. 60, 24–31. 10.1016/j.ctrv.2017.08.004 28866366

[B5] Amin R. Tripathi K. Sanderson R. D. (2020). Nuclear Heparanase Regulates Chromatin Remodeling, Gene Expression and PTEN Tumor Suppressor Function. Cells 9, 2038. 10.3390/cells9092038 PMC7564302 32899927

[B6] Aras S. Zaidi M. R. (2017). TAMeless Traitors: Macrophages in Cancer Progression and Metastasis. Br. J. Cancer 117, 1583–1591. 10.1038/bjc.2017.356 29065107 PMC5729447

[B7] Attanoos R. L. Churg A. Galateau-Salle F. Gibbs A. R. Roggli V. L. (2018). Malignant Mesothelioma and its Non-Asbestos Causes. Arch. Pathol. Lab. Med. 142, 753–760. 10.5858/arpa.2017-0365-RA 29480760

[B8] Awad M. M. Jones R. E. Liu H. Lizotte P. H. Ivanova E. V. Kulkarni M. (2016). Cytotoxic T Cells in PD-L1-Positive Malignant Pleural Mesotheliomas Are Counterbalanced by Distinct Immunosuppressive Factors. Cancer Immunol. Res. 4, 1038–1048. 10.1158/2326-6066.CIR-16-0171 27856426

[B9] Baas P. Scherpereel A. Nowak A. K. Fujimoto N. Peters S. Tsao A. S. (2021). First-Line Nivolumab Plus Ipilimumab in Unresectable Malignant Pleural Mesothelioma (CheckMate 743): a Multicentre, Randomised, Open-Label, Phase 3 Trial. Lancet 397, 375–386. 10.1016/S0140-6736(20)32714-8 33485464

[B10] Barash U. Lapidot M. Zohar Y. Loomis C. Moreira A. Feld S. (2018). Involvement of Heparanase in the Pathogenesis of Mesothelioma: Basic Aspects and Clinical Applications. J. Natl. Cancer Inst. 110, 1102–1114. 10.1093/jnci/djy032 29579286 PMC6186523

[B11] Belderbos R. A. Vroman H. Aerts. Jgjv. (2020). Cellular Immunotherapy and Locoregional Administration of CAR T-Cells in Malignant Pleural Mesothelioma. Front. Oncol. 10, 777. 10.3389/fonc.2020.00777 32582537 PMC7283907

[B13] Boyango I. Barash U. Naroditsky I. Li J. P. Hammond E. Ilan N. (2014). Heparanase Cooperates with Ras to Drive Breast and Skin Tumorigenesis. Cancer Res. 74, 4504–4514. 10.1158/0008-5472.CAN-13-2962 24970482 PMC4134691

[B14] Bueno R. Stawiski E. W. Goldstein L. D. Durinck S. De Rienzo A. Modrusan Z. (2016). Comprehensive Genomic Analysis of Malignant Pleural Mesothelioma Identifies Recurrent Mutations, Gene Fusions and Splicing Alterations. Nat. Genet. 48, 407–416. 10.1038/ng.3520 26928227

[B15] Canino C. Luo Y. Marcato P. Blandino G. Pass H. I. Cioce M. (2015). A STAT3-NFkB/DDIT3/CEBPβ axis Modulates ALDH1A3 Expression in Chemoresistant Cell Subpopulations. Oncotarget 6, 12637–12653. 10.18632/oncotarget.3703 25868979 PMC4494963

[B16] Cantini L. Hassan R. Sterman D. H. Aerts J. G. J. V. (2020). Emerging Treatments for Malignant Pleural Mesothelioma: Where Are We Heading? Front. Oncol. 10, 343. 10.3389/fonc.2020.00343 32226777 PMC7080957

[B17] Ceresoli G. L. Aerts J. G. Dziadziuszko R. Ramlau R. Cedres S. van Meerbeeck J. P. (2019). Tumour Treating Fields in Combination with Pemetrexed and Cisplatin or Carboplatin as First-Line Treatment for Unresectable Malignant Pleural Mesothelioma (STELLAR): a Multicentre, Single-Arm Phase 2 Trial. Lancet Oncol. 20, 1702–1709. 10.1016/S1470-2045(19)30532-7 31628016

[B18] Chirieac L. R. Barletta J. A. Yeap B. Y. Richards W. G. Tilleman T. Bueno R. (2013). Clinicopathologic Characteristics of Malignant Mesotheliomas Arising in Patients with a History of Radiation for Hodgkin and Non-Hodgkin Lymphoma. J. Clin. Oncol. 31, 4544–4549. 10.1200/JCO.2013.49.9616 24248693

[B19] Christoph D. C. Eberhardt W. E. (2014). Systemic Treatment of Malignant Pleural Mesothelioma: New Agents in Clinical Trials Raise hope of Relevant Improvements. Curr. Opin. Oncol. 26, 171–181. 10.1097/CCO.0000000000000053 24441503

[B20] Chu G. J. van Zandwijk N. Rasko J. E. J. (2019). The Immune Microenvironment in Mesothelioma: Mechanisms of Resistance to Immunotherapy. Front. Oncol. 9, 1366. 10.3389/fonc.2019.01366 31867277 PMC6908501

[B21] Chung Y. S. Kim M. Cha Y. J. Kim K. A. Shim H. S. (2020). Expression of V-Set Immunoregulatory Receptor in Malignant Mesothelioma. Mod. Pathol. 33, 263–270. 10.1038/s41379-019-0328-3 31363159

[B22] Conway E. Rossi F. Fernandez-Perez D. Ponzo E. Ferrari K. J. Zanotti M. (2021). BAP1 Enhances Polycomb Repression by Counteracting Widespread H2AK119ub1 Deposition and Chromatin Condensation. Mol. Cel. 81, 3526–e8. 10.1016/j.molcel.2021.06.020 PMC8428331 34186021

[B23] Correale P. Pentimalli F. Baglio G. Krstic-Demonacos M. Saladino R. E. Giordano A. (2021). Is There Already a Need of Reckoning on Cancer Immunotherapy? Front. Pharmacol. 12, 638279. 10.3389/fphar.2021.638279 33841155 PMC8033763

[B24] Corson J. M. Renshaw A. A. (1996). “Pathology of Mesothelioma,” in Comprehensive Textbook of Thoracic Oncology. Editors Aisner J. Arrigada R. Green M. R. Martini N. Perry M. C. (Baltimore (MD): Williams & Wilkins).

[B25] Cury P. M. Butcher D. N. Corrin B. Nicholson A. G. (1999). The Use of Histological and Immunohistochemical Markers to Distinguish Pleural Malignant Mesothelioma and *In Situ* Mesothelioma from Reactive Mesothelial Hyperplasia and Reactive Pleural Fibrosis. J. Pathol. 189, 251–257. 10.1002/(SICI)1096-9896(199910)189:2<251:AID-PATH412>3.0.CO;2-F 10547583

[B26] Dabir S. Kluge A. Kresak A. Yang M. Fu P. Groner B. (2014). Low PIAS3 Expression in Malignant Mesothelioma Is Associated with Increased STAT3 Activation and Poor Patient Survival. Clin. Cancer Res. 20, 5124–5132. 10.1158/1078-0432.CCR-14-1233 25124686

[B27] de Cubas A. A. Rathmell W. K. (2018). Epigenetic Modifiers: Activities in Renal Cell Carcinoma. Nat. Rev. Urol. 15, 599–614. 10.1038/s41585-018-0052-7 30030490 PMC8366903

[B28] Dhont L. Mascaux C. Belayew A. (2016). The Helicase-like Transcription Factor (HLTF) in Cancer: Loss of Function or Oncomorphic Conversion of a Tumor Suppressor? Cel. Mol. Life Sci. 73, 129–147. 10.1007/s00018-015-2060-6 PMC11108516 26472339

[B29] Edmunds J. W. Mahadevan L. C. Clayton A. L. (2008). Dynamic Histone H3 Methylation during Gene Induction: HYPB/Setd2 Mediates All H3K36 Trimethylation. EMBO J. 27, 406–420. 10.1038/sj.emboj.7601967 18157086 PMC2168397

[B30] Fahey C. C. Davis I. J. (2017). SETting the Stage for Cancer Development: SETD2 and the Consequences of Lost Methylation. Cold Spring Harb Perspect. Med. 7, a026468. 10.1101/cshperspect.a026468 28159833 PMC5411680

[B31] Feijoó E. Alfaro C. Mazzolini G. Serra P. Peñuelas I. Arina A. (2005). Dendritic Cells Delivered inside Human Carcinomas Are Sequestered by Interleukin-8. Int. J. Cancer 116, 275–281. 10.1002/ijc.21046 15800914

[B32] Fennell D. A. Ewings S. Ottensmeier C. Califano R. Hanna G. G. Hill K. Confirm trial investigators (2021). Nivolumab versus Placebo in Patients with Relapsed Malignant Mesothelioma (CONFIRM): a Multicentre, Double-Blind, Randomised, Phase 3 Trial. Lancet Oncol. 22, 1530–1540. 10.1016/S1470-2045(21)00471-X 34656227 PMC8560642

[B33] Franci G. Sarno F. Nebbioso A. Altucci L. (2017). Identification and Characterization of PKF118-310 as a KDM4A Inhibitor. Epigenetics 12, 198–205. 10.1080/15592294.2016.1249089 27767379 PMC5406213

[B34] Fu T. He Q. Sharma P. (2011). The ICOS/ICOSL Pathway Is Required for Optimal Antitumor Responses Mediated by Anti-CTLA-4 Therapy. Cancer Res. 71, 5445–5454. 10.1158/0008-5472.CAN-11-1138 21708958

[B35] Galffy G. Mohammed K. A. Nasreen N. Ward M. J. Antony V. B. (1999). Inhibition of Interleukin-8 Reduces Human Malignant Pleural Mesothelioma Propagation in Nude Mouse Model. Oncol. Res. 11, 187–194. 10566617

[B36] Galoczova M. Coates P. Vojtesek B. (2018). STAT3, Stem Cells, Cancer Stem Cells and P63. Cel. Mol. Biol. Lett. 23, 12. 10.1186/s11658-018-0078-0 PMC5863838 29588647

[B37] Gordon G. J. Dong L. Yeap B. Y. Richards W. G. Glickman J. N. Edenfield H. (2009). Four-gene Expression Ratio Test for Survival in Patients Undergoing Surgery for Mesothelioma. J. Natl. Cancer Inst. 101, 678–686. 10.1093/jnci/djp061 19401544 PMC2677573

[B38] Guanizo A. C. Fernando C. D. Garama D. J. Gough D. J. (2018). STAT3: a Multifaceted Oncoprotein. Growth Factors 36, 1–14. 10.1080/08977194.2018.1473393 29873274

[B39] Guerra-Calderas L. González-Barrios R. Herrera L. A. Cantú de León D. Soto-Reyes E. (2015). The Role of the Histone Demethylase KDM4A in Cancer. Cancer Genet. 208, 215–224. 10.1016/j.cancergen.2014.11.001 25633974

[B40] Hanahan D. Weinberg R. A. (2011). Hallmarks of Cancer: the Next Generation. Cell 144, 646–674. 10.1016/j.cell.2011.02.013 21376230

[B41] Hernández-Monge J. Rousset-Roman A. B. Medina-Medina I. Olivares-Illana V. (2016). Dual Function of MDM2 and MDMX toward the Tumor Suppressors P53 and RB. Genes Cancer 7, 278–287. 10.18632/genesandcancer.120 28050229 PMC5115168

[B42] Hmeljak J. Sanchez-Vega F. Hoadley K. A. Shih J. Stewart C. Heiman D. (2018). Integrative Molecular Characterization of Malignant Pleural Mesothelioma. Cancer Discov. 8, 1548–1565. 10.1158/2159-8290.CD-18-0804 30322867 PMC6310008

[B43] Jayatilleke K. M. Hulett M. D. (2020). Heparanase and the Hallmarks of Cancer. J. Transl Med. 18, 453. 10.1186/s12967-020-02624-1 33256730 PMC7706218

[B44] Khan M. W. Saadalla A. Ewida A. H. Al-Katranji K. Al-Saoudi G. Giaccone Z. T. (2018). The STAT3 Inhibitor Pyrimethamine Displays Anti-cancer and Immune Stimulatory Effects in Murine Models of Breast Cancer. Cancer Immunol. Immunother. 67, 13–23. 10.1007/s00262-017-2057-0 28875329 PMC5783191

[B45] Klose R. J. Yamane K. Bae Y. Zhang D. Erdjument-Bromage H. Tempst P. (2006). The Transcriptional Repressor JHDM3A Demethylates Trimethyl Histone H3 Lysine 9 and Lysine 36. Nature 442, 312–316. 10.1038/nature04853 16732292

[B46] Lapidot M. Barash U. Vlodavsky I. Pass H. (2018). Heparanase Inhibitors Restrain Mesothelioma. Oncotarget 9, 36830–36832. 10.18632/oncotarget.26243 30627323 PMC6305150

[B47] Lapidot M. Case A. E. Larios D. Gandler H. I. Meng C. Tosic I. (2020a). Inhibitors of the Transcription Factor STAT3 Decrease Growth and Induce Immune Response Genes in Models of Malignant Pleural Mesothelioma (MPM). Cancers (Basel) 13, 7. 10.3390/cancers13010007 PMC7792575 33374980

[B48] Lapidot M. Gill R. R. Mazzola E. Freyaldenhoven S. Swanson S. J. Jaklitsch M. T. (2020b). Pleurectomy Decortication in the Treatment of Malignant Pleural Mesothelioma: Encouraging Results and Novel Prognostic Implications Based on Experience in 355 Consecutive Patients. Ann. Surg. 10.1097/SLA.0000000000004306 33278174

[B49] Lapidot M. Case A. E. Weisberg E. L. Meng C. Walker S. R. Garg S. (2021). Essential Role of the Histone Lysine Demethylase KDM4A in the Biology of Malignant Pleural Mesothelioma (MPM). Br. J. Cancer 125, 582–592. 10.1038/s41416-021-01441-7 34088988 PMC8368004

[B50] Lee H. S. Jang H. J. Choi J. M. Zhang J. de Rosen V. L. Wheeler T. M. (2018). Comprehensive Immunoproteogenomic Analyses of Malignant Pleural Mesothelioma. JCI Insight 3, e98575. 10.1172/jci.insight.98575 PMC5928857 29618661

[B51] Liou D. Z. Berry M. F. (2018). Diagnosis and Management of Mesothelioma. AME Med. J. 3, 99. 10.21037/amj.2018.09.11

[B52] Maio M. Scherpereel A. Calabrò L. Aerts J. Perez S. C. Bearz A. (2017). Tremelimumab as Second-Line or Third-Line Treatment in Relapsed Malignant Mesothelioma (DETERMINE): a Multicentre, International, Randomised, Double-Blind, Placebo-Controlled Phase 2b Trial. Lancet Oncol. 18, 1261–1273. 10.1016/S1470-2045(17)30446-1 28729154

[B53] Martin T. D. Patel R. S. Cook D. R. Choi M. Y. Patil A. Liang A. C. (2021). The Adaptive Immune System Is a Major Driver of Selection for Tumor Suppressor Gene Inactivation. Science 373, 1327–1335. 10.1126/science.abg5784 34529489 PMC13397623

[B54] Matsuzaki H. Maeda M. Lee S. Nishimura Y. Kumagai-Takei N. Hayashi H. (2012). Asbestos-induced Cellular and Molecular Alteration of Immunocompetent Cells and Their Relationship with Chronic Inflammation and Carcinogenesis. J. Biomed. Biotechnol. 2012, 492608. 10.1155/2012/492608 22500091 PMC3304550

[B55] Monaghan L. Massett M. E. Bunschoten R. P. Hoose A. Pirvan P. A. Liskamp R. M. J. (2019). The Emerging Role of H3K9me3 as a Potential Therapeutic Target in Acute Myeloid Leukemia. Front. Oncol. 9, 705. 10.3389/fonc.2019.00705 31428579 PMC6687838

[B56] Nicolini F. Bocchini M. Bronte G. Delmonte A. Guidoboni M. Crinò L. (2019). Malignant Pleural Mesothelioma: State-Of-The-Art on Current Therapies and Promises for the Future. Front. Oncol. 9, 1519. 10.3389/fonc.2019.01519 32039010 PMC6992646

[B57] Noy R. Pollard J. W. (2014). Tumor-Associated Macrophages: from Mechanisms to Therapy. Immunity 41, 49–61. 10.1016/j.immuni.2014.06.010 25035953 PMC4137410

[B58] Pencik J. Pham H. T. Schmoellerl J. Javaheri T. Schlederer M. Culig Z. (2016). JAK-STAT Signaling in Cancer: From Cytokines to Non-Coding Genome. Cytokine 87, 26–36. 10.1016/j.cyto.2016.06.017 27349799 PMC6059362

[B59] Pfister S. X. Markkanen E. Jiang Y. Sarkar S. Woodcock M. Orlando G. (2015). Inhibiting WEE1 Selectively Kills Histone H3K36me3-Deficient Cancers by dNTP Starvation. Cancer Cel. 28, 557–568. 10.1016/j.ccell.2015.09.015 PMC4643307 26602815

[B60] Popat S. Curioni-Fontecedro A. Dafni U. Shah R. O'Brien M. Pope A. (2020). A Multicentre Randomised Phase III Trial Comparing Pembrolizumab versus Single-Agent Chemotherapy for Advanced Pre-treated Malignant Pleural Mesothelioma: the European Thoracic Oncology Platform (ETOP 9-15) PROMISE-Meso Trial. Ann. Oncol. 31, 1734–1745. 10.1016/j.annonc.2020.09.009 32976938

[B61] Remon J. Reguart N. Corral J. Lianes P. (2015). Malignant Pleural Mesothelioma: new hope in the Horizon with Novel Therapeutic Strategies. Cancer Treat. Rev. 41, 27–34. 10.1016/j.ctrv.2014.10.007 25467107

[B62] Roca Suarez A. A. Van Renne N. Baumert T. F. Lupberger J. (2018). Viral Manipulation of STAT3: Evade, Exploit, and Injure. Plos Pathog. 14, e1006839. 10.1371/journal.ppat.1006839 29543893 PMC5854428

[B63] Schulz-Heddergott R. Stark N. Edmunds S. J. Li J. Conradi L. C. Bohnenberger H. (2018). Therapeutic Ablation of Gain-Of-Function Mutant P53 in Colorectal Cancer Inhibits Stat3-Mediated Tumor Growth and Invasion. Cancer Cel. 34, 298–e7. 10.1016/j.ccell.2018.07.004 PMC6582949 30107178

[B64] Shteingauz A. Boyango I. Naroditsky I. Hammond E. Gruber M. Doweck I. (2015). Heparanase Enhances Tumor Growth and Chemoresistance by Promoting Autophagy. Cancer Res. 75, 3946–3957. 10.1158/0008-5472.CAN-15-0037 26249176 PMC4573896

[B65] Sugarbaker D. J. Norberto J. J. Bueno R. (1997). Current Therapy for Mesothelioma. Cancer Control 4, 326–334. 10.1177/107327489700400404 10763038

[B66] Szczepanski A. P. Wang L. (2021). Emerging Multifaceted Roles of BAP1 Complexes in Biological Processes. Cel. Death Discov. 7, 20. 10.1038/s41420-021-00406-2 PMC7822832 33483476

[B67] Takakura A. Nelson E. A. Haque N. Humphreys B. D. Zandi-Nejad K. Frank D. A. (2011). Pyrimethamine Inhibits Adult Polycystic Kidney Disease by Modulating STAT Signaling Pathways. Hum. Mol. Genet. 20, 4143–4154. 10.1093/hmg/ddr338 21821671 PMC3188991

[B12] Tan Y. C. Srivastava S. Won B. M. Kanteti R. Arif Q. Husain A. N. (2019). EPHA2 Mutations With Oncogenic Characteristics in Squamous Cell Lung Cancer and Malignant Pleural Mesothelioma. Oncogenesis 8, 49. 10.1038/s41389-019-0159-6 31484920 PMC6726628

[B68] Vlodavsky I. Singh P. Boyango I. Gutter-Kapon L. Elkin M. Sanderson R. D. (2016). Heparanase: From Basic Research to Therapeutic Applications in Cancer and Inflammation. Drug Resist. Updat. 29, 54–75. 10.1016/j.drup.2016.10.001 27912844 PMC5447241

[B69] Wald O. Sugarbaker D. J. (2018). New Concepts in the Treatment of Malignant Pleural Mesothelioma. Annu. Rev. Med. 69, 365–377. 10.1146/annurev-med-041316-085813 29029582

[B70] Wang L. Chang J. Varghese D. Dellinger M. Kumar S. Best A. M. (2013). A Small Molecule Modulates Jumonji Histone Demethylase Activity and Selectively Inhibits Cancer Growth. Nat. Commun. 4, 2035. 10.1038/ncomms3035 23792809 PMC3724450

[B71] Wilson B. G. Wang X. Shen X. McKenna E. S. Lemieux M. E. Cho Y. J. (2010). Epigenetic Antagonism between Polycomb and SWI/SNF Complexes during Oncogenic Transformation. Cancer Cell 18, 316–328. 10.1016/j.ccr.2010.09.006 20951942 PMC2957473

[B72] Xiang M. Kim H. Ho V. T. Walker S. R. Bar-Natan M. Anahtar M. (2016). Gene Expression-Based Discovery of Atovaquone as a STAT3 Inhibitor and Anticancer Agent. Blood 128, 1845–1853. 10.1182/blood-2015-07-660506 27531676 PMC5054697

[B73] Yang H. Hall S. R. R. Sun B. Zhao L. Gao Y. Schmid R. A. (2021). NF2 and Canonical Hippo-YAP Pathway Define Distinct Tumor Subsets Characterized by Different Immune Deficiency and Treatment Implications in Human Pleural Mesothelioma. Cancers (Basel) 13, 1561. 10.3390/cancers13071561 33805359 PMC8036327

[B74] Yang H. Berezowska S. Dorn P. Zens P. Chen P. Peng R.-W. (2022). Tumor-infiltrating Lymphocytes Are Functionally Inactivated by CD90+ Stromal Cells and Reactivated by Combined Ibrutinib and Rapamycin in Human Pleural Mesothelioma. Theranostics 12, 167–185. 10.7150/thno.61209 34987640 PMC8690914

[B75] Yoshikawa Y. Emi M. Hashimoto-Tamaoki T. Ohmuraya M. Sato A. Tsujimura T. (2016). High-density Array-CGH with Targeted NGS Unmask Multiple Noncontiguous Minute Deletions on Chromosome 3p21 in Mesothelioma. Proc. Natl. Acad. Sci. U S A. 113, 13432–13437. 10.1073/pnas.1612074113 27834213 PMC5127333

[B76] Young L. C. Hendzel M. J. (2013). The Oncogenic Potential of Jumonji D2 (JMJD2/KDM4) Histone Demethylase Overexpression. Biochem. Cel Biol 91, 369–377. 10.1139/bcb-2012-0054 24219278

[B77] Yuan W. Xie J. Long C. Erdjument-Bromage H. Ding X. Zheng Y. (2009). Heterogeneous Nuclear Ribonucleoprotein L Is a Subunit of Human KMT3a/Set2 Complex Required for H3 Lys-36 Trimethylation Activity *In Vivo* . J. Biol. Chem. 284, 15701–15707. 10.1074/jbc.M808431200 19332550 PMC2708867

[B78] Zalcman G. Mazieres J. Margery J. Greillier L. Audigier-Valette C. Moro-Sibilot D. (2016). Bevacizumab for Newly Diagnosed Pleural Mesothelioma in the Mesothelioma Avastin Cisplatin Pemetrexed Study (MAPS): a Randomised, Controlled, Open-Label, Phase 3 Trial. Lancet 387, 1405–1414. 10.1016/s0140-6736(15)01238-6 26719230

[B79] Zhang M. Luo J. L. Sun Q. Harber J. Dawson A. G. Nakas A. (2021). Clonal Architecture in Mesothelioma Is Prognostic and Shapes the Tumour Microenvironment. Nat. Commun. 12, 1751. 10.1038/s41467-021-21798-w 33741915 PMC7979861

[B80] Zhu X. Sun L. Lan J. Xu L. Zhang M. Luo X. (2016). BRG1 Targeting STAT3/VEGFC Signaling Regulates Lymphangiogenesis in Colorectal Cancer. Oncotarget 7, 36501–36509. 10.18632/oncotarget.9038 27145366 PMC5095016

